# Vibration dataset of main journal bearings in internal combustion engines under diverse climatic and varying operating conditions

**DOI:** 10.1016/j.dib.2024.111214

**Published:** 2024-12-07

**Authors:** Muhammad Noman Riaz, Amir Hamza, Hamid Jabbar, Manzar Abbas, Mohsin Islam Tiwana

**Affiliations:** a Department of Mechatronics Engineering, College of Electrical & Mechanical Engineering, National University of Sciences & Technology, Islamabad, Pakistan; b Department of Avionics Engineering, Air University, Aerospace and Aviation Campus Kamra, Islamabad, Pakistan

**Keywords:** Internal combustion engine, Main journal bearings, Vibration signatures, Environmental condition

## Abstract

The dataset includes vibration signatures from both healthy and faulty main journal bearings of an internal combustion engine, captured with a tri-axial accelerometer mounted on the bearings' housing. The engine was exposed to various climatic and operating conditions, including variations in temperature and humidity, and tested at different engine rotation speeds, following a standard, MIL-STD-810G. The data was collected in a state-of-the-art climatic and vibration chamber to simulate real-world environmental conditions. The dataset comprises more than 500 files, each recorded under a range of climatic and operational conditions, with root mean square (RMS) values included. This dataset provides a valuable and reliable benchmark for researchers and practitioners to validate their diagnostic algorithms and models against real engine conditions, as no such dataset is publicly available.

Specifications Table

**Table:** 

Subject	Mechanical Engineering.
Specific subject area	Automotive Engine Journal Bearings Condition Monitoring
Type of data	Graph Raw, Processed.
Data collection	Automobile engine bearings were exposed to vibrations under varying climatic and operating conditions, with the engine placed inside a climate-controlled chamber. A tri-axial accelerometer with a sensitivity of 100 mV/g was mounted on the housing of the engine bearing being tested. Additionally, feedback and force sensors were attached to a custom-built iron fixture to allow for real-time adjustments to the vibration signal. The experiments followed a Full-Factorial Design, evaluating both healthy and faulty bearings. Data collection and analysis were conducted using the VR9500 Vibration Controller in combination with the VibrationVIEW software.
Data source location	Department of Mechatronics Engineering, College of Electrical and Mechanical Engineering, National University of Sciences and Technology, Islamabad, Pakistan.
Data accessibility	Repository name: Mendeley Data Data identification number: 10.17632/3fcrrdjjvk.5 Direct URL to data: https://data.mendeley.com/datasets/3fcrrdjjvk/5 Instructions for accessing these data: Access the website
Related research article	‘none’.

## 1 Value of the Data

• This dataset provides vibration signals from automobile internal combustion engine main journal bearings (both healthy and faulty) tested under various temperature, humidity, engine rotation speed, and vibration conditions, following MIL-STD-810G Method 520.3 titled, *“Temperature, Humidity, Vibration and Altitude”*. The dataset uniquely includes environmental conditions, making it a valuable benchmark for comparing the performance of fault detection and identification techniques in realistic scenarios, particularly for engines operating in urban driving conditions or stop-and-go traffic.
• The data is ideal for machine learning and deep learning algorithms, offering insights into both time and frequency domain characteristics of engine bearings under dynamic conditions. This enables its use in predictive maintenance and condition monitoring studies, helping to prevent catastrophic failures.
• With its versatility, the dataset can be used alone or combined with others to improve fault diagnosis accuracy through Transfer Learning. Collected under diverse climatic and operational conditions, it ensures generalizability of models, distinguishing it from datasets focused solely on industrial bearings.
• Although the engine's size or rotation per minute (RPM) may be lower than typical automotive engines, the dataset is highly valuable for studying smaller engines such as scooters, motorcycles, or small commercial vehicles. It offers unique insights into fuel efficiency, reliability, and wear in low-RPM engines, making it applicable to regions like South Asia, Far-East Asia, Latin America, and Africa, where such engines are prevalent.

## 2 Background

Over the last five decades, the monitoring of engine vibration conditions has emerged as a significant avenue for enhancing prominence across a diverse range of industries. Engine vibration analysis stands out as a highly effective technology for condition-based maintenance [1]. The primary contributor to vibrations in internal combustion engines is often traced back to bearing faults [2]. Internal combustion (IC) engines are used as energy converters in a variety of applications, including automobiles, equipment, power generation, and the movement of people and commodities [3]. Due to their advantageous key properties, including their high power-to-weight ratio, robustness, efficacy, affordability, and the availability of large-scale fuel supply infrastructure, they are widely used [4]. With continued usage of the engine, the issue will worsen day by day, substantially impairing performance and even causing the engine to stop working, which could lead to a traffic collision or something else dangerous [5].

One of the most critical parts of IC engines are engine bearings. The unpredictable operating circumstances of the engine, such as frequent starts and stops, the presence of high temperatures and pressures, and load and speed changes, frequently subject engine bearings to wear [6]. Researchers face a challenging task in diagnosing faults in engine components, given the intricacies involved in engine operations. The emergence of faults in engine components leads to a subsequent decline in performance and an increase in maintenance costs.

This study specifically focuses on smaller engines commonly used in urban environments and low-speed driving conditions, where low RPM is standard. A detailed explanation in the subsequent paragraphs of why this dataset focuses on low-RPM engines used in urban traffic conditions, particularly in regions like South-Asia, South-East-Asia, Far-East Asia, Latin America, and Africa. This justification highlights the importance of studying smaller engines that operate in congested, low-speed environments, which is relevant to the real-world applications of the dataset. In addition, with reference to geographical relevance, we have expanded on the specific geographical regions where smaller engine-powered vehicles are prevalent, emphasizing that the dataset is targeted at vehicles commonly used in these regions. The following points highlighted the practical significance and the prospective acceptance in global market.

• **Climatic and Traffic Conditions**: The selected environmental conditions (temperature and humidity) are representative of regions like South-East Asia, South-Asia, Far-East Asia, Latin America, and Africa, where climatic variability is significant. These regions frequently experience hot climates, high humidity, and seasonal extremes that can impact vehicle performance.
• **Low RPM and Traffic Congestion**: The low RPM values (1000, 1500, 2000) were specifically chosen to reflect urban driving conditions prevalent in these regions, where traffic congestion is common. Vehicles often operate at lower RPMs due to frequent stops and slow-moving traffic. In addition, these lower RPM levels are typical of smaller engines, which are more prevalent in these regions, where fuel efficiency and affordability are prioritized. The smaller engine sizes (e.g., 660cc) and low-speed driving habits result in different vibration and wear patterns, which this dataset captures.
• **Relevance to Smaller Engines**: In regions like South-Asia, Africa, and Latin America, smaller vehicles such as scooters, motorcycles, and small commercial vehicles dominate the transportation landscape. These vehicles often operate at low RPMs due to urban infrastructure and road conditions, making the selected RPM range highly relevant for studying fuel efficiency, engine reliability, and bearing wear in these environments.
• **Climatic Variations**: The temperature and humidity values (−10 °C to 45 °C and 0% to 100% humidity) reflect the range of climatic conditions that vehicles in these regions encounter, from freezing temperatures in some areas to high temperatures and humidity in others, especially in tropical regions. These conditions help test the durability of engine components under real-world stresses.
• **Testing for Harsh Conditions**: By combining temperature, humidity, and vibration conditions, the dataset simulates the challenges faced by engines in regions with unpredictable climates and heavy traffic, where stop-and-go driving and low-speed operation are common. This makes the dataset ideal for studying the combined effects of environmental factors and operational conditions on engine performance and reliability.

To develop effective fault diagnosis algorithms, it is essential to acquire vibration pattern data from engine bearings while replicating a range of climatic (temperature and humidity) and terrain conditions (surface and road discontinuities) using state-of-the-art climatic and vibration chamber as vibration patterns, influenced by climatic (temperature, humidity) and terrain conditions (altitude, surface), are vital for accurate condition monitoring. The dataset, collected according to MIL-STD-810G, accurately simulates real-world environmental conditions, providing relevant and precise data for tailoring diagnostic solutions.

As compare to the other research works [[7], [8], [9], [10], [11], [12], [13], [14], [15], [16]] that focused solely on the laboratory controlled environment, this work presents a more holistic approach as the multiple environmental and operating stressors are simultaneously acting on the engine/ device under test (DUT) which is not explored in such a detailed manner previously. This dataset contributes significantly to the research community by offering a unique resource for developing and validating machine learning and deep learning models for fault detection and predictive maintenance in low-RPM engines. The data, collected under real-world environmental conditions, enables researchers to simulate and study the wear patterns of journal bearings in urban environments. Additionally, this dataset opens up opportunities for transfer learning applications, where models trained on this data can be fine-tuned for other engine types.

Furthermore, emphasizing the unique aspects of this dataset compared to others, this dataset distinguishes itself from existing similar datasets through its focus on low-RPM engines in real-world conditions, which are commonly found in urban environments across South-Asia, South-East-Asia, Far-East Asia, Africa and Latin America. Unlike many datasets that focus on high-RPM industrial or automotive engines, this dataset was collected following MIL-STD-810G standards under varying environmental factors such as temperature, humidity, and vibration, replicating real-world operational stressors. Furthermore, it includes both healthy and faulty bearings data, offering a comprehensive resource for developing fault detection and predictive maintenance models. This highlights the uniqueness of our work and explains how it fills a gap in publicly available data.

Summarily, the chosen values for temperature, humidity, RPM, and other conditions are based on the real-world usage of smaller, fuel-efficient vehicles in regions where low RPM driving and harsh environmental conditions prevail. This makes the dataset uniquely suited for studies focusing on urban driving conditions and smaller engines, which are common in regions like South-Asia, Far-East Asia, Latin America, and Africa.

## 3 Data Description

The data was gathered using a tri-axial vibration sensor connected to a VR9500 vibration controller, which supports up to 128 channels, offering significant flexibility for test setups ranging from simple to highly complex configurations. The VR9500 controller operates through **VibrationVIEW** software, known for its user-friendly interface and advanced functionality for real-time test design and monitoring. The collected data is presented as Word files as well as .csv files generated by the **VibrationVIEW** software installed on the VR9500 vibration controller. These files contain various graphical representations, including ***Acceleration Spectral Density*** graphs, ***Drive Signal*** graphs, ***Acceleration Waveforms****,* ***Transmissibility graphs****,* ***Acceleration* vs *Time graphs***, and ***Channel Measurements***, which display processed root mean square (**RMS)** values for each axis, given that a tri-axial sensor was employed for data acquisition. The unit of vibration measurement across the three axes is gravitational acceleration **g** (1 g ≈ 9.8665 m/s²).

***Raw data*** refers to the direct time-domain acceleration signals recorded by the accelerometer attached to the engine bearing housing or system. This data captures instantaneous acceleration values at each moment. The ***RMS*** value provides a statistical measure of the overall signal strength over time. RMS is computed from the raw time-domain data, offering a single value representing the energy of the vibration over a given period. The RMS values obtained from the VR9500 vibration controller are derived from the raw acceleration data but are classified as processed data since they represent the average magnitude over time. This makes RMS particularly valuable for quantifying vibration levels consistently and simply. The channels displaying RMS values under the ***Channel Measurement*** section of the report correspond to the axes of the accelerometer, which are typically aligned to different axes of vibration (e.g., radial, axial, and tangential directions relative to the bearing). The RMS values of each channel provide insight into the behavior of the system along these respective axes.

**Fig. 1:**
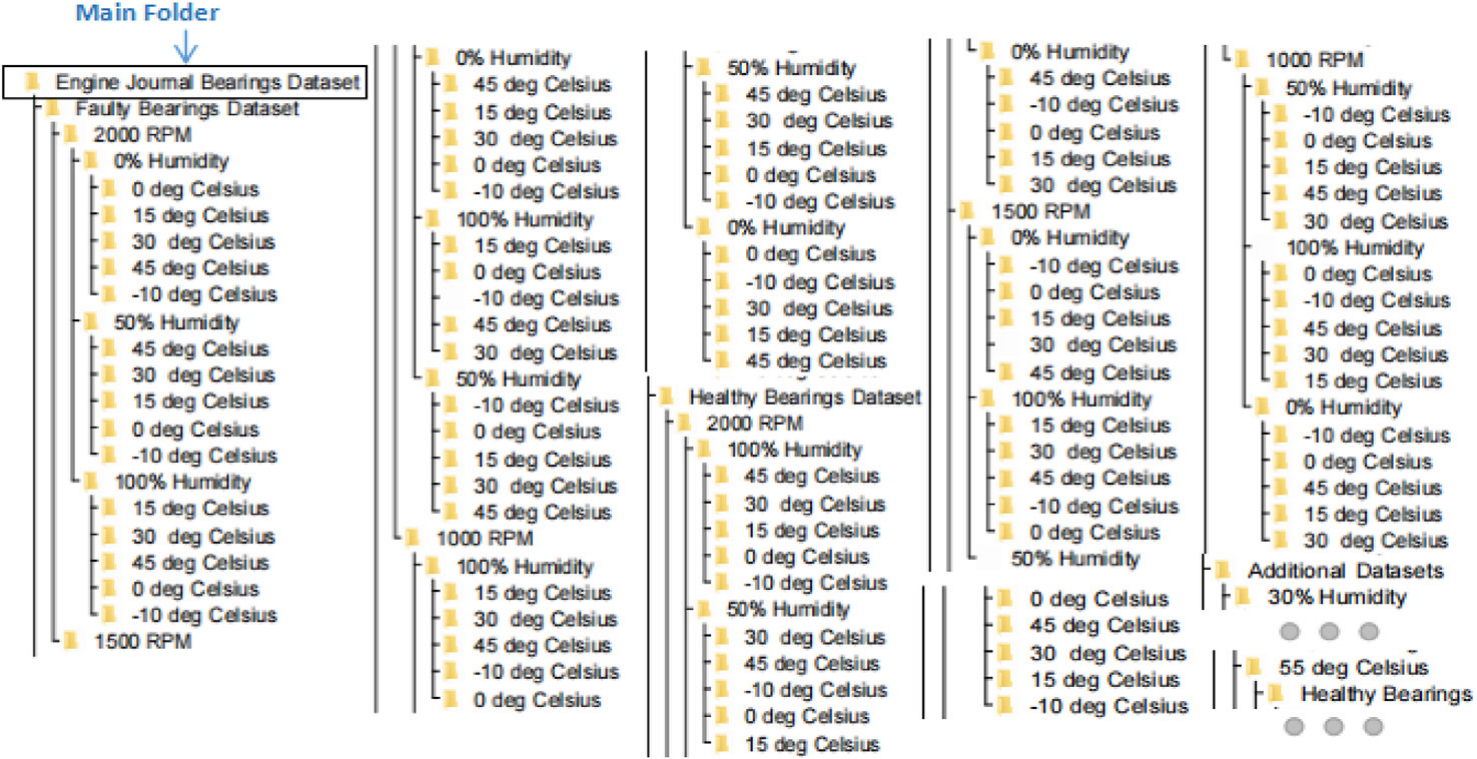
Hierarchy/ directory of engine bearings dataset.

The files and reports generated by the **VibrationVIEW** software are structured in a specific format, as outlined in Fig. 1. These files share the same base name as the folder they are located in, with folders categorized by bearing condition (healthy / faulty), engine rotation speed in rotations per minute (RPM), humidity, and temperature. The acquired dataset is systematically organized into a folder structure, as shown in Fig. 1. To minimize storage requirements, the dataset has been compressed into a .zip format. The entire dataset is divided into five distinct hierarchical layers. In the first layer, there are three main folders titled: ‘Additional Dataset’, ‘Faulty Bearings Dataset’, and ‘Healthy Bearings Dataset’. The second layer within the ‘Faulty Bearings Dataset’ and ‘Healthy Bearings Dataset’ is organized by engine rotation speed values (1000 RPM, 1500 RPM, 2000 RPM). Moving to the third layer, the data is further categorized by three humidity levels: 0%, 50%, and 100%. In the fourth layer, the data is divided across five different temperature levels: −10 °C, 0 °C, 15 °C, 30 °C, and 45 °C.

Additionally, the ‘Additional Dataset’ folder is split into two sub-folders: ‘30% Humidity’ and ‘55 Deg’. Within the second layer of these sub-folders, the data is separated into ‘Healthy Bearings’ and ‘Faulty Bearings’. The third layer of the ‘Additional Dataset’ categorizes the data by RPM values, followed by a division based on temperature. For the ‘55 Deg’ sub-folder, the data is first categorized by engine rotation speed, and then further subdivided into four humidity levels: 0%, 30%, 60%, and 100%, forming the final layer. Similar data structure has been adopted for dataset available in .csv file format. Similar data hierarchical structure is adopted for .csv files available in separate .zip folder at the data repository.

The dataset consists of more than 500 files/ reports, documenting the conditions of both healthy and faulty bearings, which were tested under varying climatic and operational conditions. Additional experiments were conducted each for healthy and faulty bearings at 30% Humidity level across temperature levels ranging from −20 °C to 50 °C at engine rotation speed of 1000, 1500, and 2000 rpm as well as at 55 °C while varying the humidity levels from 0 to 100% (0,30,60, 100%) and engine rotation speed of 1000, 1500, and 2000 RPM to provide additional dataset for researchers/ practitioners for validation of their models.

**Fig. 2:**
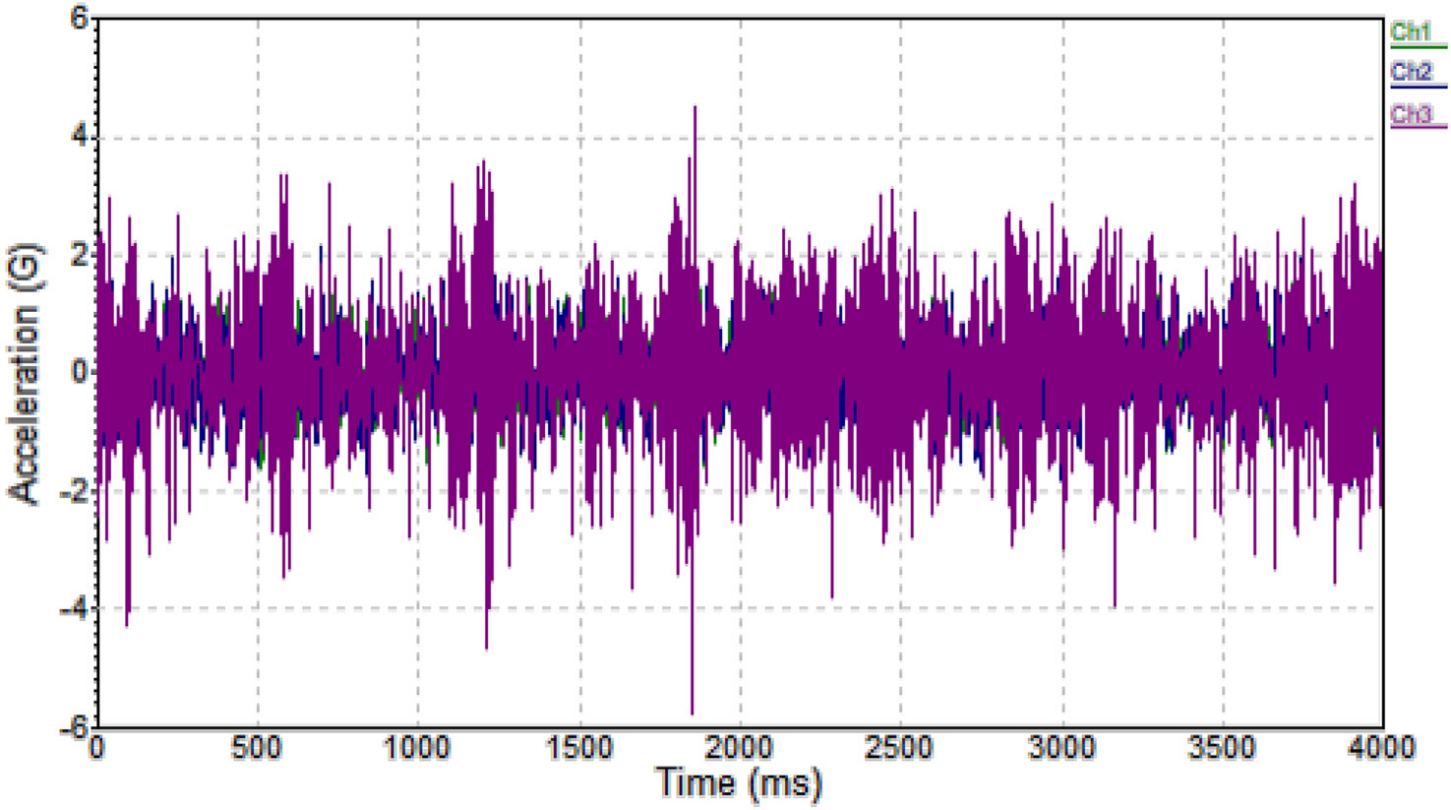
Acceleration Waveform.
• The **Drive Signal graph** (Fig 3) represents the **voltage density** applied to the system as it changes with frequency. This energy input is transmitted to the engine bearings, simulating real-world conditions. By correlating the **drive signal** with the **vibration response** (e.g., RMS values), we can observe how different energy levels at specific frequencies affect bearing performance. For example, faults in bearings may exhibit increased vibration amplitudes at certain drive signal frequencies, helping identify resonance or wear-related issues. This correlation is vital for diagnosing faults and understanding bearing behavior under varying loads and environmental conditions.

In the subsequent paragraphs of this section, the key figures, tables, and summary statistics that reflect the most significant findings from the dataset have been presented. These additions help illustrate the diversity of conditions (such as temperature, RPM, and humidity) under which the data was collected. It is ensured that the manuscript contains enough sample data to demonstrate the value of the dataset, while the full dataset remains available in the public repository for further analysis. The presentation of the data is as follows:

• Acceleration Waveform
  The acceleration waveform graph (Fig. 2) communicates the acceleration experienced by the DUT for a snapshot of time. Specifically it is the acceleration value that is being measured at the exact point where the accelerometer is mounted on the DUT at a specific moment in time. The **Acceleration vs. Time graph (Acceleration Waveform)** displays the bearing's vibration response as acceleration changes over time. This waveform captures transient events, allowing the identification of **irregularities or spikes** that may indicate faults such as bearing wear, misalignment, or imbalance. By analyzing the **peak acceleration values** over time, we can determine how the bearings react to sudden loads or operational changes. Faulty bearings typically exhibit higher or more frequent spikes in the waveform, making this graph essential for detecting early signs of failure in real-time.

**Fig. 3:**
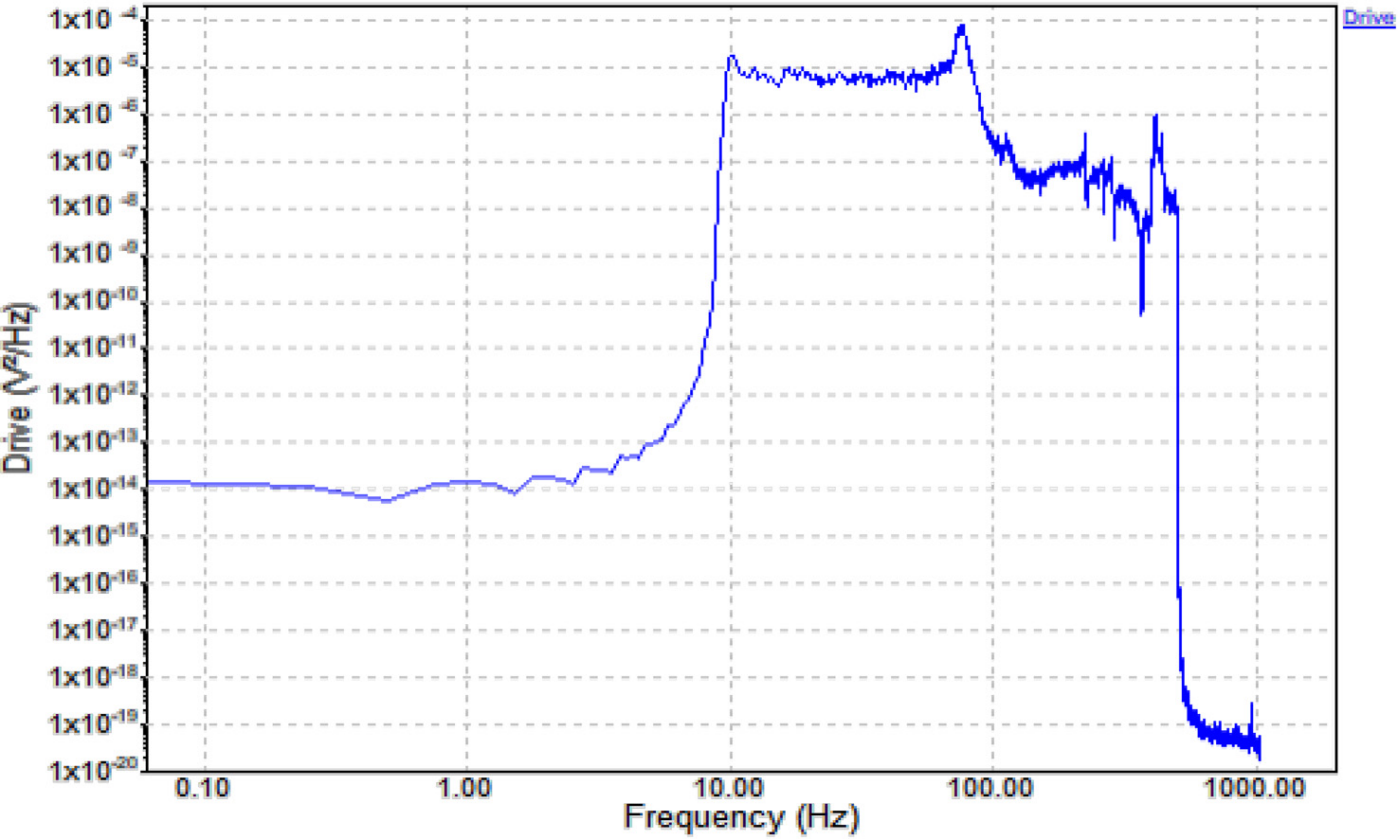
Drive Acceleration Graph.
• The **Acceleration Spectral Density (ASD) graph** (Fig. 4) represents the distribution of vibration energy across different frequencies. It provides insights into how engine bearings respond to varying frequency inputs, helping identify **resonance** or **vibration peaks** that may be associated with faults such as wear or imbalance. Higher peaks at certain frequencies indicate that more energy is concentrated in those ranges, potentially signaling abnormal bearing behavior. The ASD graph is essential for diagnosing frequency-specific issues, making it a critical tool in detecting and monitoring bearing faults.

**Fig. 4:**
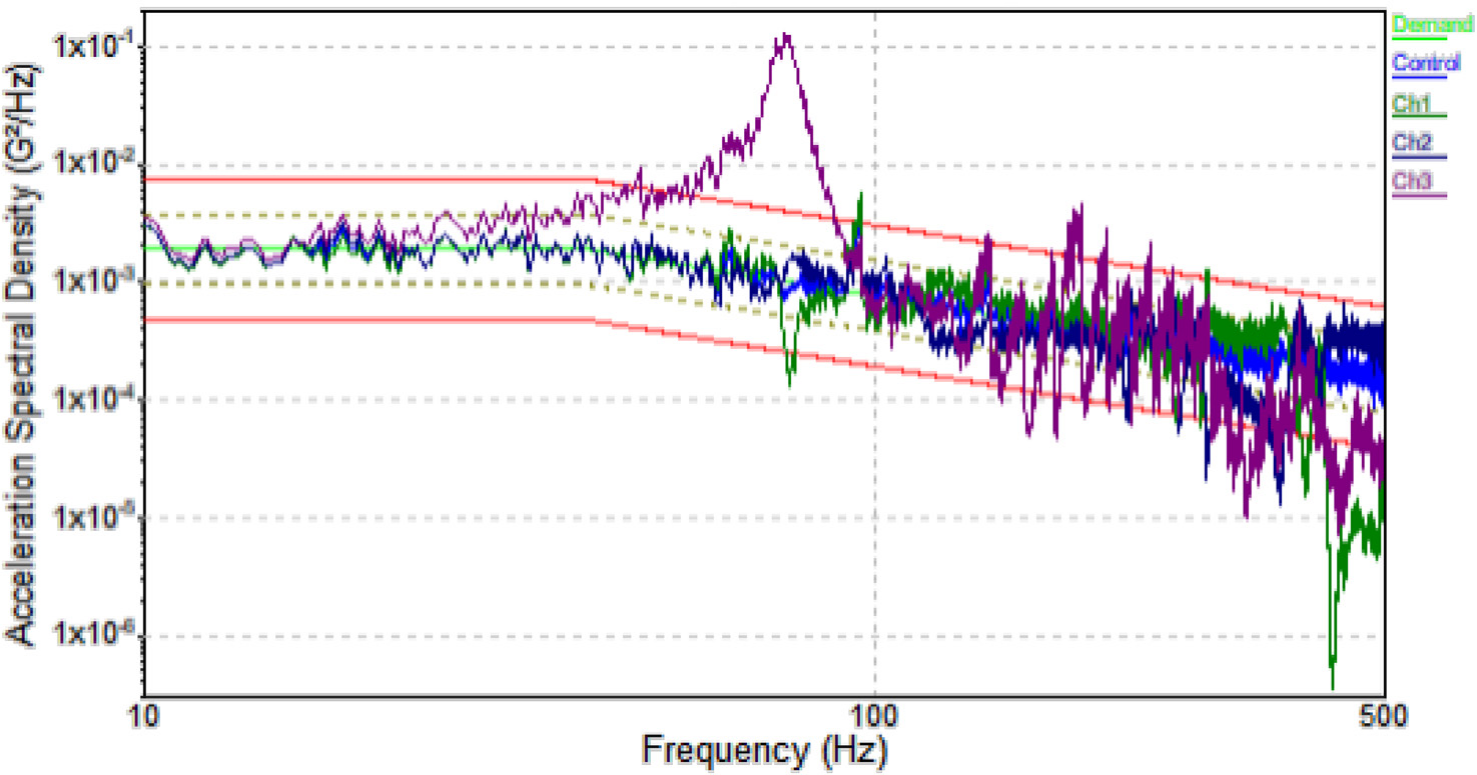
Acceleration Spectral density Graph.
• The **Transmissibility Graph** (Fig. 5) represents the ratio of output to input vibration, showing how much vibration energy is transmitted through the system. This graph is crucial for understanding how **vibrations from the drive signal** or external forces are passed through the bearings and engine components. High transmissibility values at certain frequencies indicate that a large portion of the vibration is being transmitted, potentially leading to **resonance** or **amplified stress** on the bearings. By analyzing this graph, we can assess the **efficiency of vibration isolation** and detect issues like **bearing wear, misalignment**, or **structural weaknesses** that allow excessive vibration to pass through the system.

**Fig. 5:**
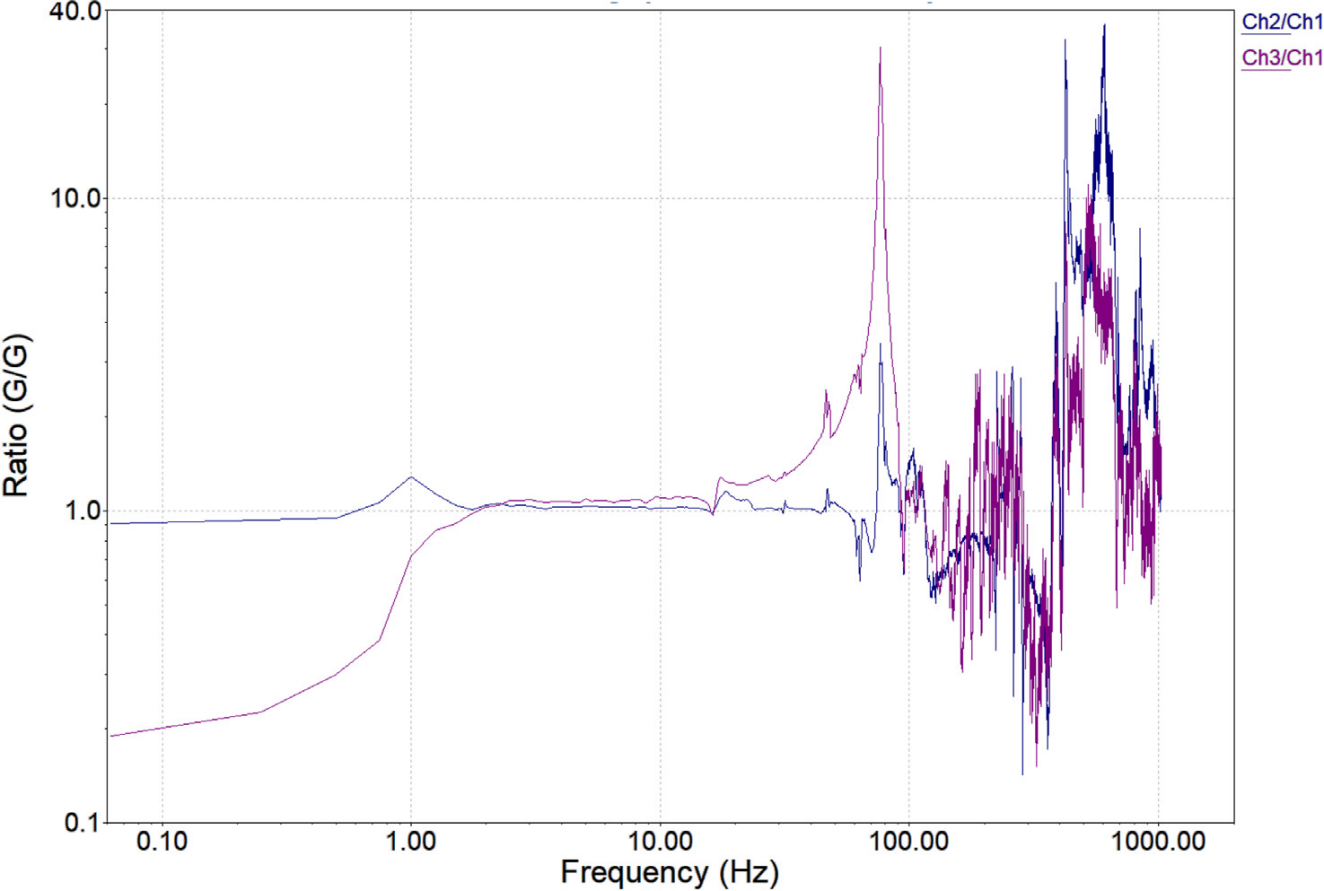
Transmissibility Graph.
• The **Acceleration vs. Time Graph** (Fig. 6) illustrates the vibration response of the bearings as acceleration changes over time. This graph is critical for detecting **transient events** such as sudden spikes, irregularities, or repeated patterns in acceleration that may indicate faults like **bearing wear, misalignment**, or **imbalances**. By observing how the acceleration behaves over time, we can identify the **severity and frequency** of abnormal events, which is essential for early fault detection and real-time monitoring of the system's health.

**Fig. 6:**
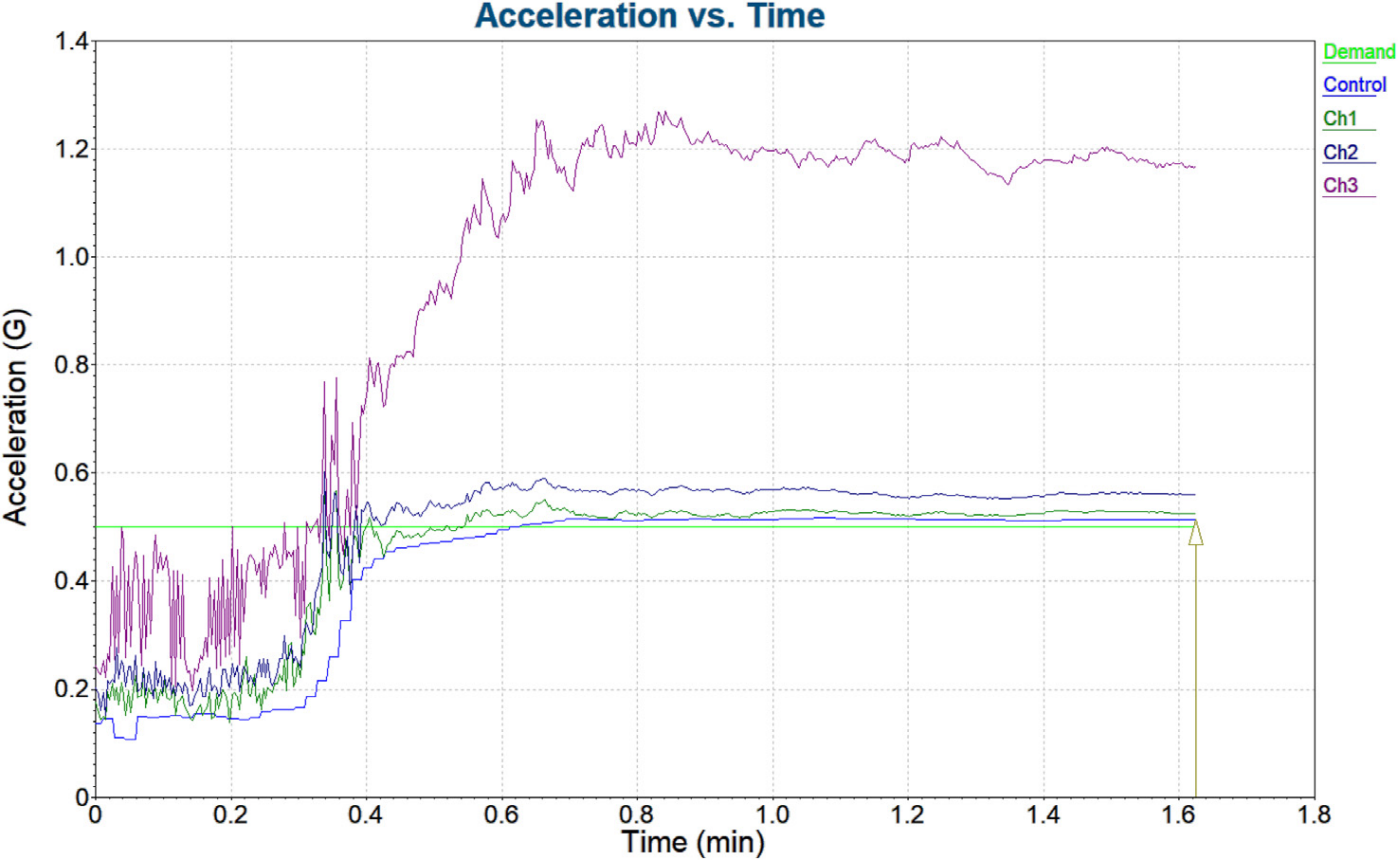
Acceleration vs. Time Graph.
• The **Drive vs. Time Graph** (Fig. 7) represents how the **voltage or input signal** applied to the system changes over time. This graph shows the magnitude and variation of the force or energy transmitted to the engine bearings during the test. By analyzing this graph alongside the **vibration response**, one can determine how changes in the input energy affect the bearing's performance. Sudden changes in the drive signal may correlate with spikes or anomalies in the vibration data, helping identify stress points that lead to bearing wear or faults.

**Fig. 7:**
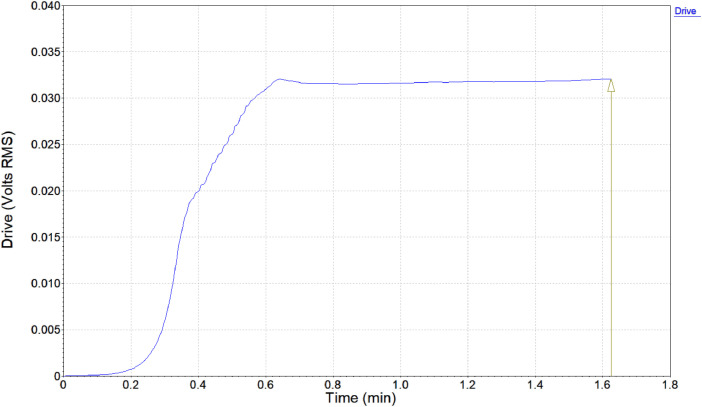
Drive vs. Time Graph.
• The **Channel Measurements** provide a summary of the **RMS (Root Mean Square)** values for each channel, capturing the overall vibration energy in the system. For the selected sample report excerpt as shown in Table 1:

**Table 1:** Summary of Channel Measurements.

Channel Measurements		
Channel Number	(Overall)	(In-Band)
Ch1	0.5237 G RMS	0.5157 G RMS
Ch2	0.5597 G RMS	0.5073 G RMS
Ch3	1.166 G RMS	1.169 G RMS
Ch4	1.715e-15 G RMS	1.479e-15 G RMS
• **Ch1** represents the overall vibration response on the bearing housing, showing **0.5237 G RMS** overall and **0.5157 G RMS** in-band, indicating consistent vibration levels in this channel.
• **Ch2** measures the **force applied to the system**, with **0.5597 G RMS** overall and **0.5073 G RMS** in-band, reflecting the input force.
• **Ch3**, representing the **vibration measured on the bearing housing**, records **1.166 G RMS** overall and **1.169 G RMS** in-band, illustrating the actual vibration experienced by the bearing.
• **Ch4**, measuring residual signals, shows very low values (near zero), indicating minimal residual vibration.

By analyzing the RMS values across channels, we can observe how the **drive and force inputs** (Ch2) relate to the **vibration response** in the bearing housing (Ch3). The higher vibration levels in Ch3 indicate the significant impact of forces on the bearings, providing key insights for condition monitoring.

**Fig. 8:**
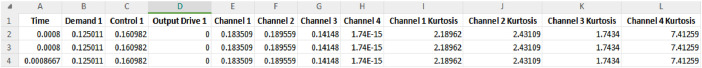
Glimpse of Vibration Dataset File in .csv Format.

In addition, the VibraionVIEW software produces a raw vibration data in .csv format. The glimpse of one of the .csv files is as shown in Fig 8. The dataset captured in this file includes multiple key parameters used to monitor and analyze the behavior of engine bearings under specific test conditions.

Each column in the dataset serves a unique purpose:

• **Time:** The first column of .csv file **r**epresents the time stamps corresponding to each recorded data point.
• **Demand 1 & Control 1:** These columns represent the input control signals used to maintain the required operational conditions, such as engine rotation speed or temperature levels.
• **Output & Drive 1:** These columns reflect the actual output drive signal applied to the engine bearings, that can be correlated with the vibration responses from the channels to assess how external forces impact the performance of the engine bearings.
• **Channels 1 to 4:** These columns depict the vibration data acquired by the attached sensors(feedback, drive, accelerometer) attached to different parts of the engine and bearing housing (Ch 3). Each channel specific vibration signals, providing insights into how different components respond under various operating and climatic conditions.
• **Kurtosis for Channels 1 to 4:** Kurtosis is a statistical measure that reflects the sharpness or flatness of the vibration signal distribution. Elevated Kurtosis values often indicate the presence of sudden, impulsive events, which could signal potential faults like cracks or bearing wear.

The combination of time-domain and statistical features like Kurtosis allows researchers to develop and refine predictive maintenance models and anomaly detection algorithms.

Therefore, the collected data provides critical insights into the vibrational characteristics of engine journal bearings through several key graphical representations, including Acceleration Spectral Density (ASD) graphs, drive signal graphs, and acceleration waveforms. These graphs help track the vibration response under different operational conditions and excitation forces, providing valuable information for fault detection and condition monitoring. Time-domain waveforms detect transient events like bearing faults, while Transmissibility graphs assess the efficiency of vibration energy transfer. Acceleration vs. Time graphs offer continuous monitoring of vibration levels, and Root Mean Square (RMS) values simplify overall vibration energy representation, making them useful for predictive maintenance.

The data plays a vital role in analyzing fault progression and journal bearing health, with a specific focus on low-RPM engines common in regions such as South Asia, Southeast Asia, Far East Asia, Africa, and Latin America. It further explores the influence of environmental factors, such as temperature and humidity, on bearing performance, ensuring real-world reliability. Collected under MIL-STD-810G standards, the dataset is unique and non-reproducible, providing invaluable resources for researchers seeking to validate machine learning models or simulate real-world bearing performance. Additionally, the document offers foundational data for training machine learning and anomaly detection models, especially in predictive maintenance and fault diagnosis applications.

## 4 Experimental Design, Materials and Methods

The subsequent paragraphs will describe the climatic chamber used to simulate environmental conditions, the passenger car engine under test, the accelerometer used to capture vibration signals from the engine bearings, and the VibrationVIEW software for analysis. After covering the tools and equipment used in the experiment, a detailed overview of the testing procedure will be provided.

### 4.1 Experimental engine and Setup

**Fig. 9:**
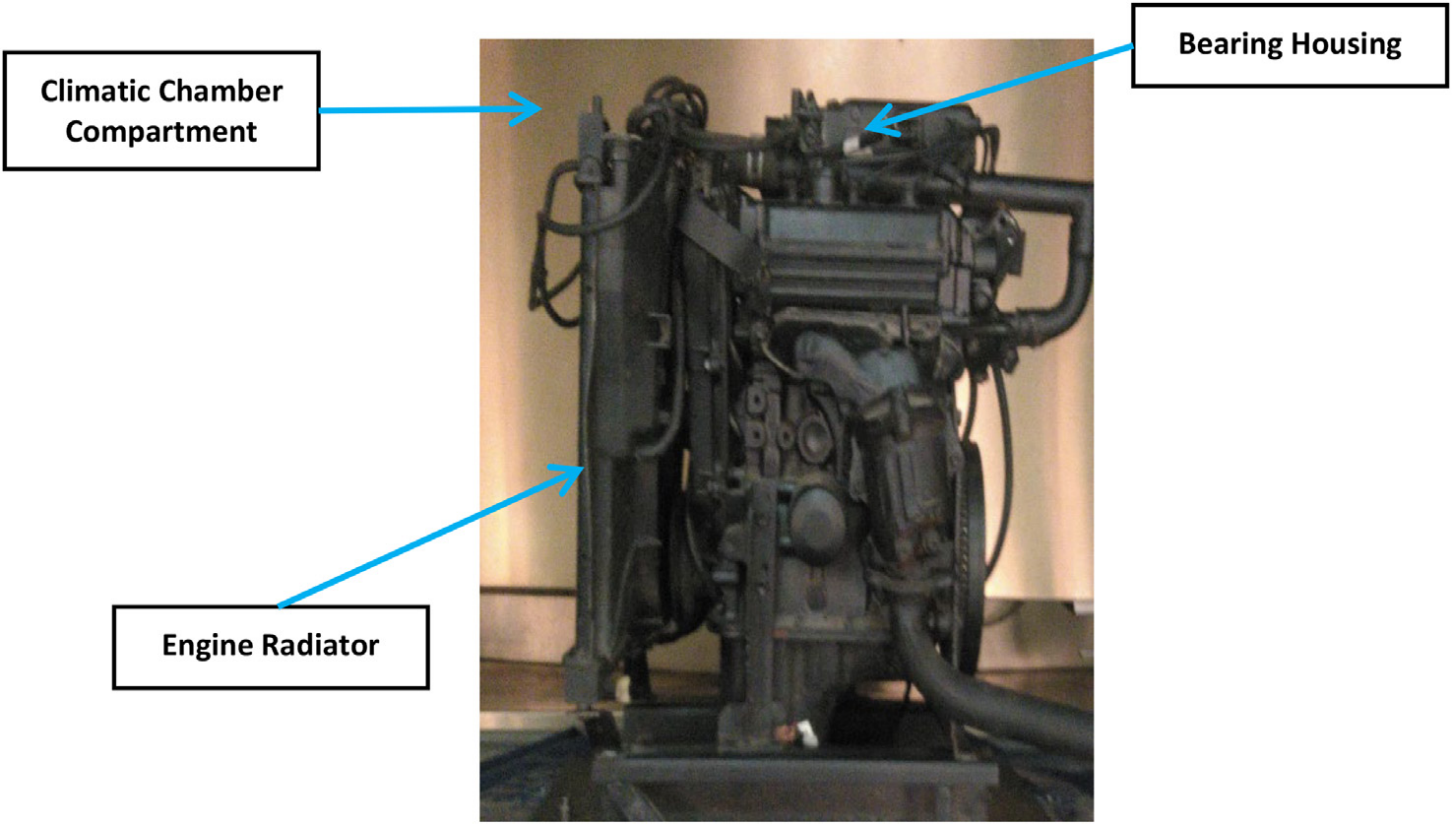
The automobile engine 658 cc.

Engine Specifications: Experimental testing was carried out on a passenger car engine with a 3-cylinder DOHC 12-valve configuration and a displacement of 658 cc, as shown in Fig. 9. This engine, which can produce up to 39 horsepower at 6500 RPM and 56 Nm of torque at 4000 RPM, is commonly used in various passenger cars and light commercial vehicles due to its mechanical efficiency. Detailed technical specifications of the engine are listed in Table 2.

**Table 2:** Main technical specifications of automobile engine.

Position/ Parameter	Description
Engine Type	DOHC 12 Valve
Engine Displacement	658cc
Fuel Type	Petrol
Compression Ratio	10: 01
Bore x Stroke	64.0 × 68.2
Engine Power	39 hp @ 6500 RPM
Torque	56 NM @ 6500 RPM
Cylinders	03
Transmission	Automatic
Fuel Capacity (Litres)	27 L
Gear Box	5 Speed

### 4.2 Climatic chamber (Environmental Simulation)

**Fig. 10:**
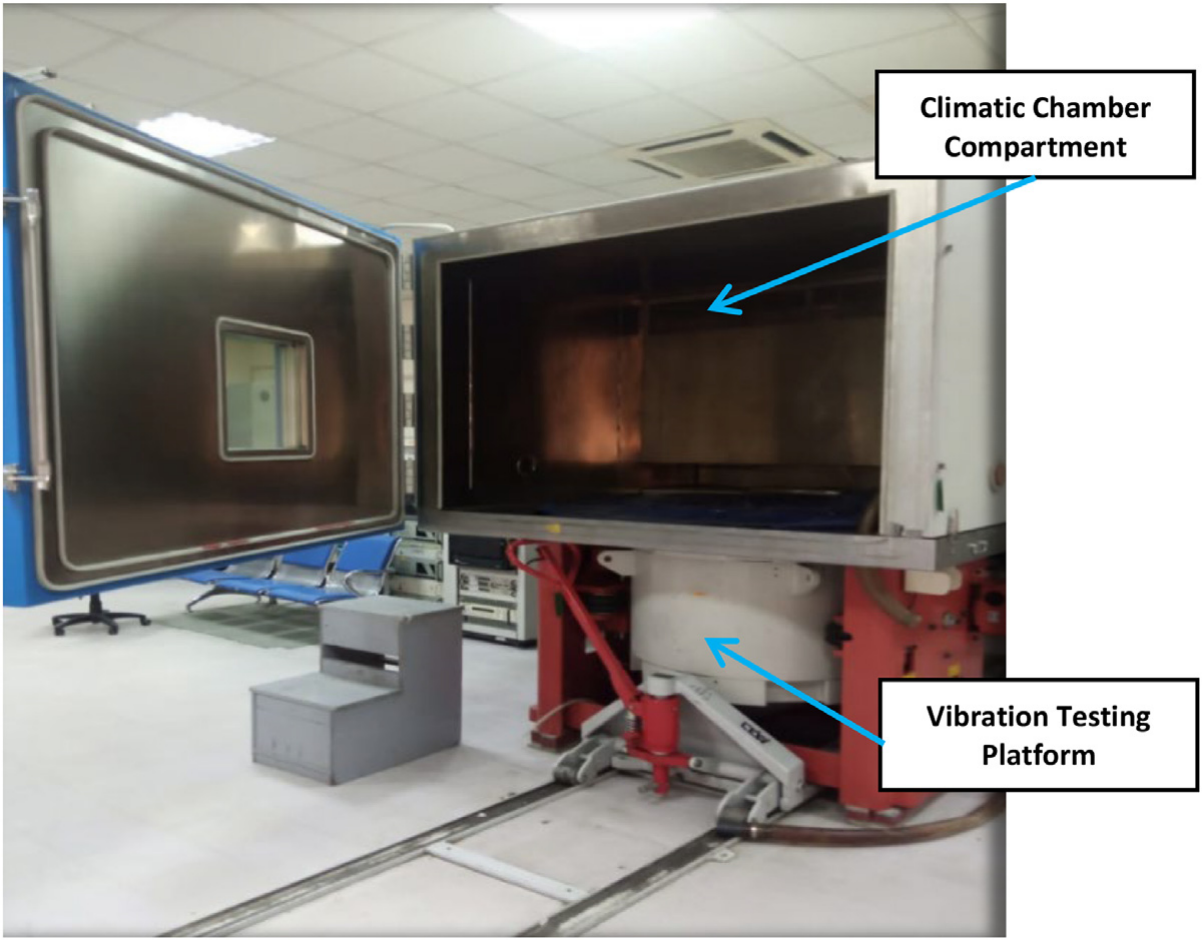
The climatic fast ramp chamber.

Chamber Specifications: The climatic chamber (Ch2800) from ACS Angelantoni, Italy, as depicted in Fig. 10, is designed to simulate a wide range of climatic and terrain conditions, including temperature, humidity, thermal, and vibration shocks. Measuring 1000 × 1010 × 1020 mm, it features a single climatic compartment capable of temperatures ranging from −70 °C to 180 °C and relative humidity up to 100%. Control and data acquisition instruments were housed in a separate room adjacent to the chamber. Technical specifications are provided in Table 3.

**Table 3:** Main technical specifications of climatic chamber.

Position/ Parameter	Description
Temp Range	−70 ∼ +180 dg C
Capacity	1030 Lit
Internal Dimensions	1000*1010*1020
Temperature Precision	± 0.5 ∼1 dg C
Heating Speed	15 dg C per min
Cooling Speed	15 dg C per min
Relative Humidity Range	10% ∼ 100%
Humidity Precision	± 3% ∼ ± 5%
Noise	80 db
Temp Range	−70 ∼ +180 dg C
Capacity	1030 Liter

Temperature and Humidity Control: The temperature was controlled using the climatic chamber, and a calibrated thermocouple sensor within the chamber was used to measure the temperature continuously during each experimental run. The sensor was calibrated to ensure a high degree of accuracy. Humidity levels were controlled using the climatic chamber and varied between 30% and 90% RH. A humidity sensor continuously monitored the levels, and its calibration ensured accurate readings, thereby capturing the effects of environmental variability.

### 4.3 Vibration data measurement

**Fig. 11:**
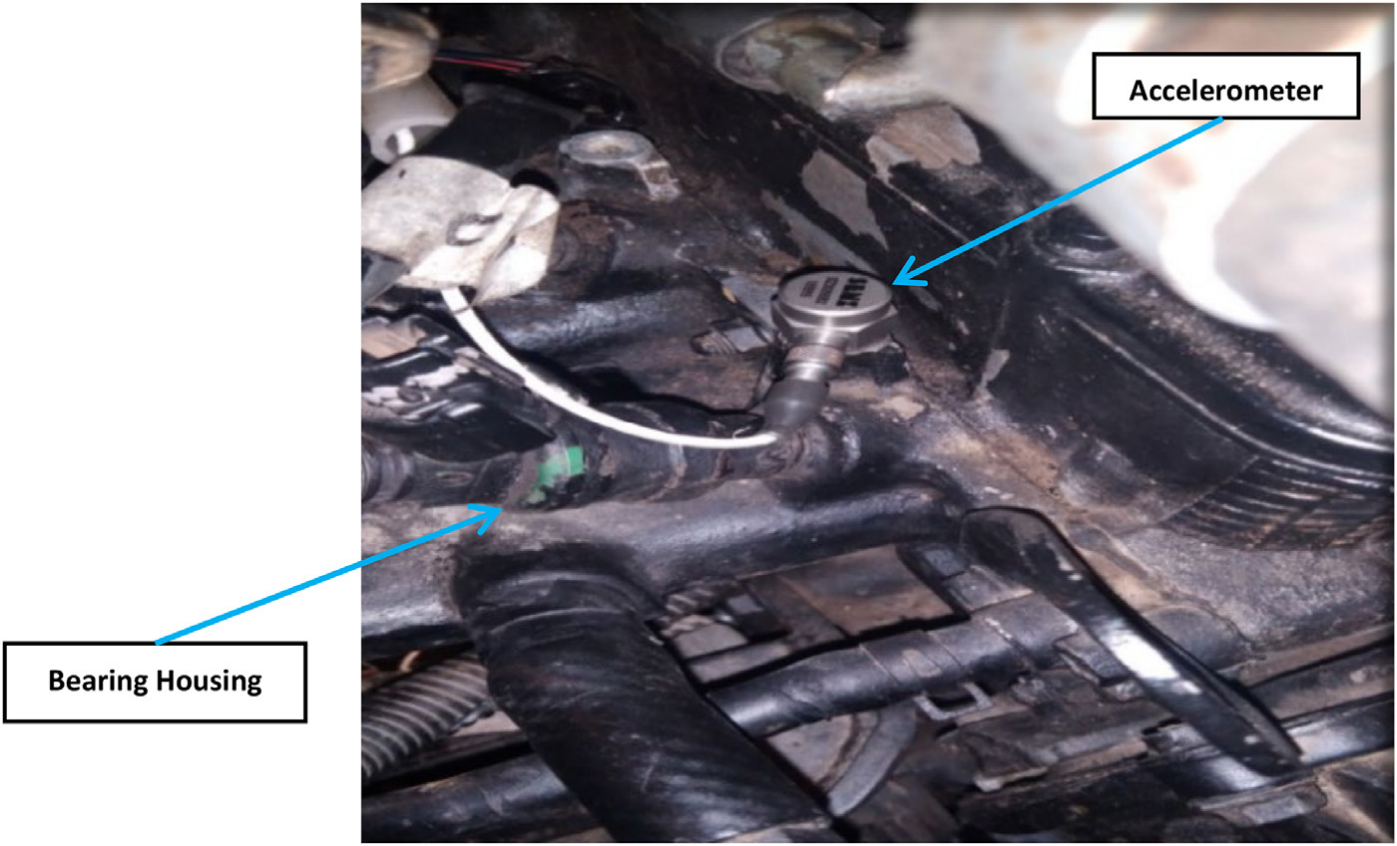
Accelerometer measuring engine bearings’ vibration signal.

**Accelerometer Used**: A PCB piezoelectric accelerometer with a sensitivity of 100 mV/G was used to measure the vibration signals from the engine bearings. The accelerometer was securely mounted on the main engine bearing housing with wax and screws, as shown in Fig. 11. Accurate vibration measurement depends on the accelerometer being positioned as close to the vibrating component as possible, and the accelerometer's sensitivity to sudden changes in vibration frequencies underscores the importance of a stable mounting to ensure precise data capture [18].

**Mounting Method**: The accelerometer was mounted directly on the bearing housing using a secure clamp to minimize any external movement or noise that could affect the measurements. Proper mounting is crucial for ensuring that the vibrations being measured are solely due to bearing performance, without influence from extraneous factors.

**Fig. 12:**
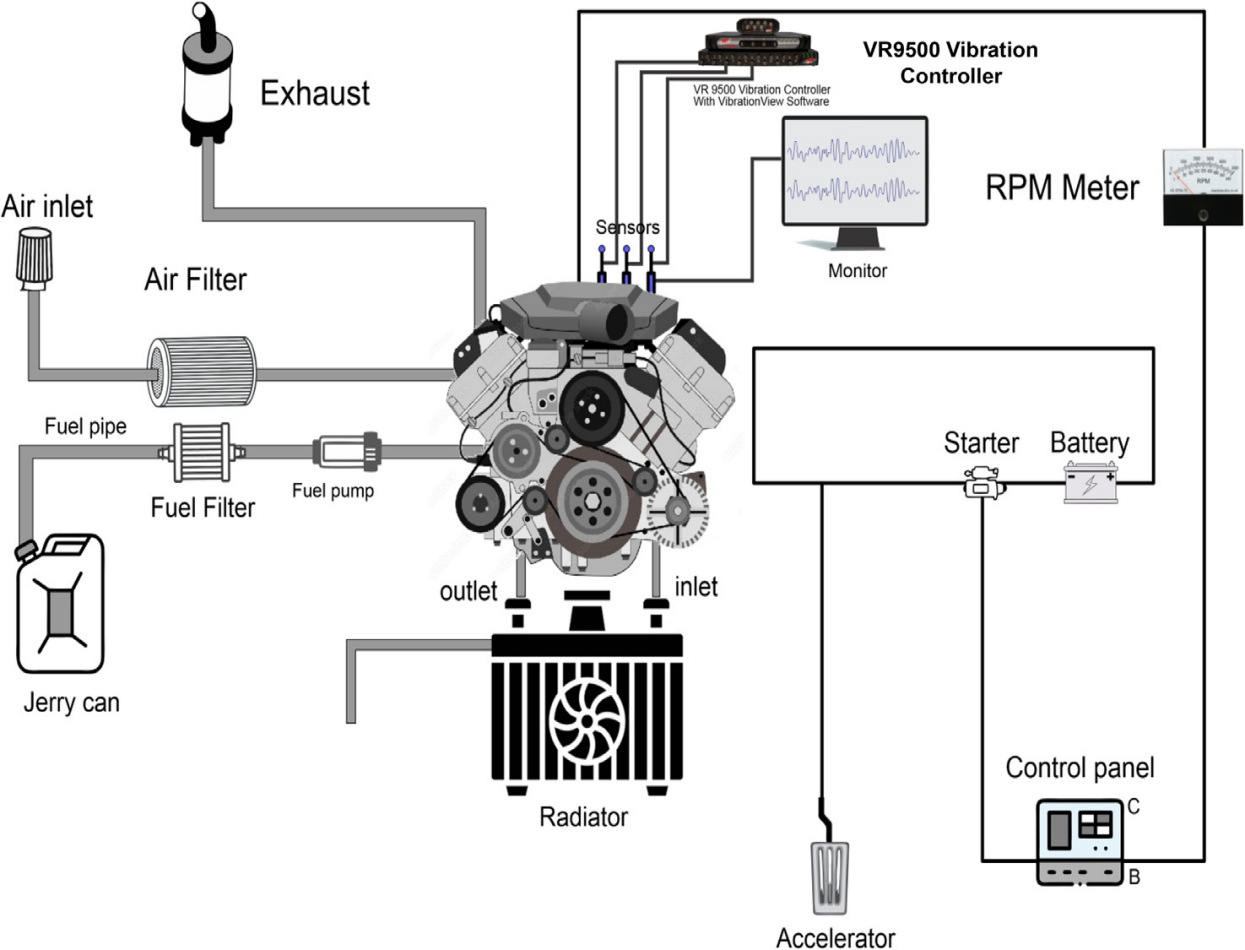
Schematic Diagram of engine testbed and data acquisition.

**Data Acquisition and Processing**: Vibration signals were transmitted via cables to a data controller and subsequently analyzed using VibrationVIEW software. The VR9500 Vibration Controller, in conjunction with the VibrationVIEW software, was used for data presentation and analysis. The VR9500 supports up to 128 channels and has a simultaneous sampling rate of 200 kHz. During data acquisition, tests were conducted at a frequency of 3.2 kHz, effectively capturing the vibration signals for condition monitoring. The processed data included time-domain acceleration waveforms, which show changes in acceleration over time, and frequency-domain data in the form of Acceleration Spectral Density (ASD) graphs. The RMS values of the acceleration signal were, in addition, calculated to provide an overall measure of vibration energy, which is a key indicator of bearing health. The experimental setup is illustrated in Fig. 12.

### 4.4 Engine speed (RPM) measurement

**Instrument Used**: A tachometer was used to measure the engine speed (RPM) during testing. The optical tachometer provides a non-contact measurement method, which was chosen to minimize interference with the engine's operation.

**RPM Control and Stability**: The throttle was used to maintain the desired engine speed during testing, ensuring stable operation at specific RPM levels—such as 1000, 1500, and 2000 RPM. The tachometer readings were continuously monitored to ensure that the engine speed was maintained consistently during data collection. Any minor fluctuations were documented to ensure complete transparency of the testing conditions.

### 4.5 Experimental procedure overview

**Fig. 13:**
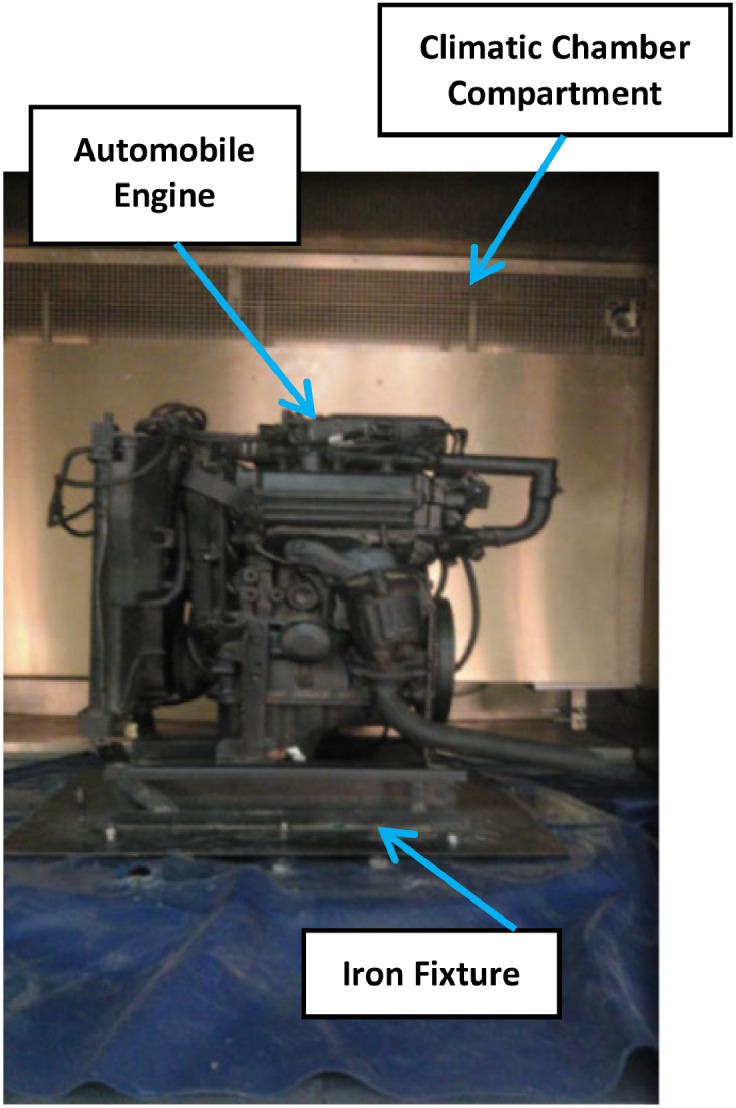
The engine fixed on chamber inside chamber.

**Fig. 14:**
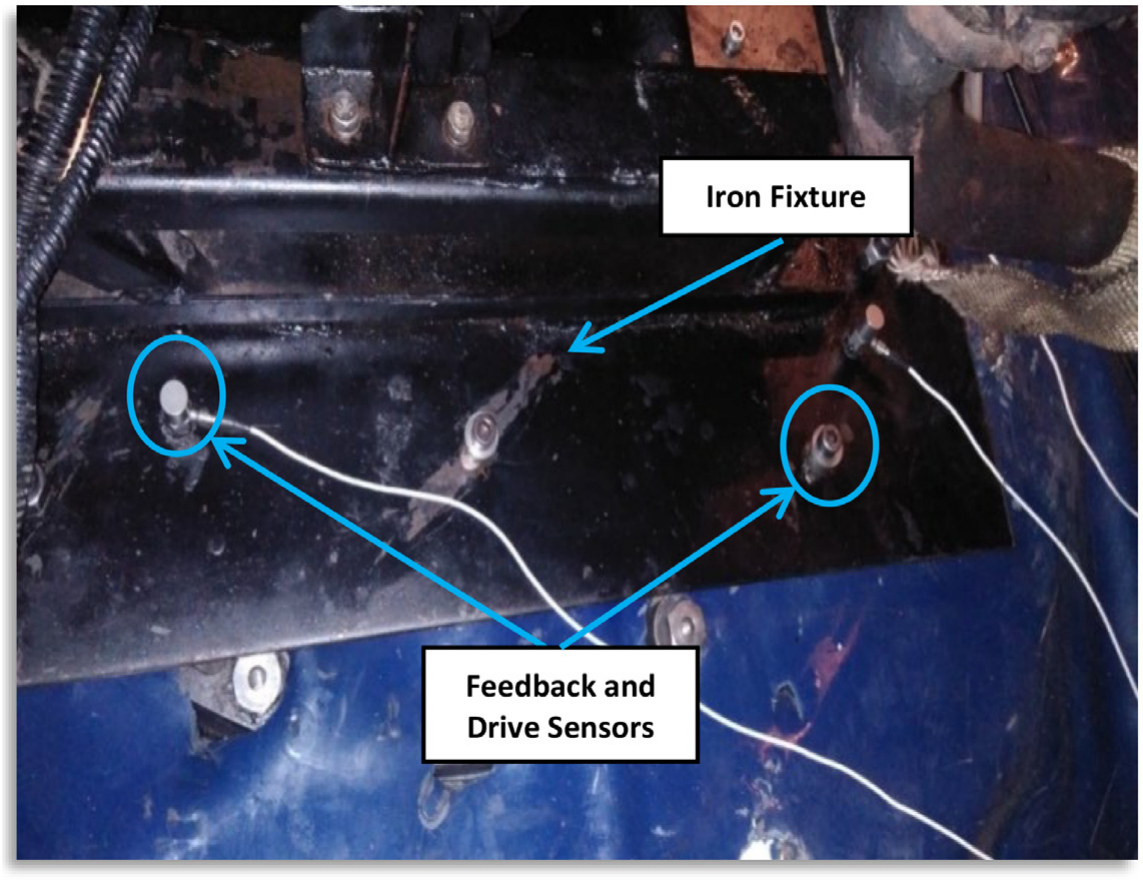
Placement of drive and feedback sensor on fixture inside chamber.

**Healthy Bearings Testing**: Initially, experiments were conducted with healthy bearings, recording the Root Mean Square (RMS) values under various climatic and operating conditions. The engine was placed in the climatic chamber and subjected to external vibration signals to simulate real-world surface and road discontinuities. It was securely mounted on a custom-designed hardened iron fixture, as shown in Fig. 13, and subjected to a 0.5 g vibration signal to replicate road conditions. A feedback sensor on the fixture provided real-time adjustments to the vibration signal, as depicted in Fig. 14.

**Faulty Bearings Testing**: After completing the experiments with healthy bearings, faulty bearings were installed, and similar experiments were conducted to acquire vibration signatures for approximately eight hours to simulate real-world conditions. Before initiating experiments with both healthy and faulty bearings, skilled technicians from an approved service center performed a thorough visual inspection. The engine was overhauled, and the bearing components were examined to prevent signal misinterpretation.

**Fig. 15:**
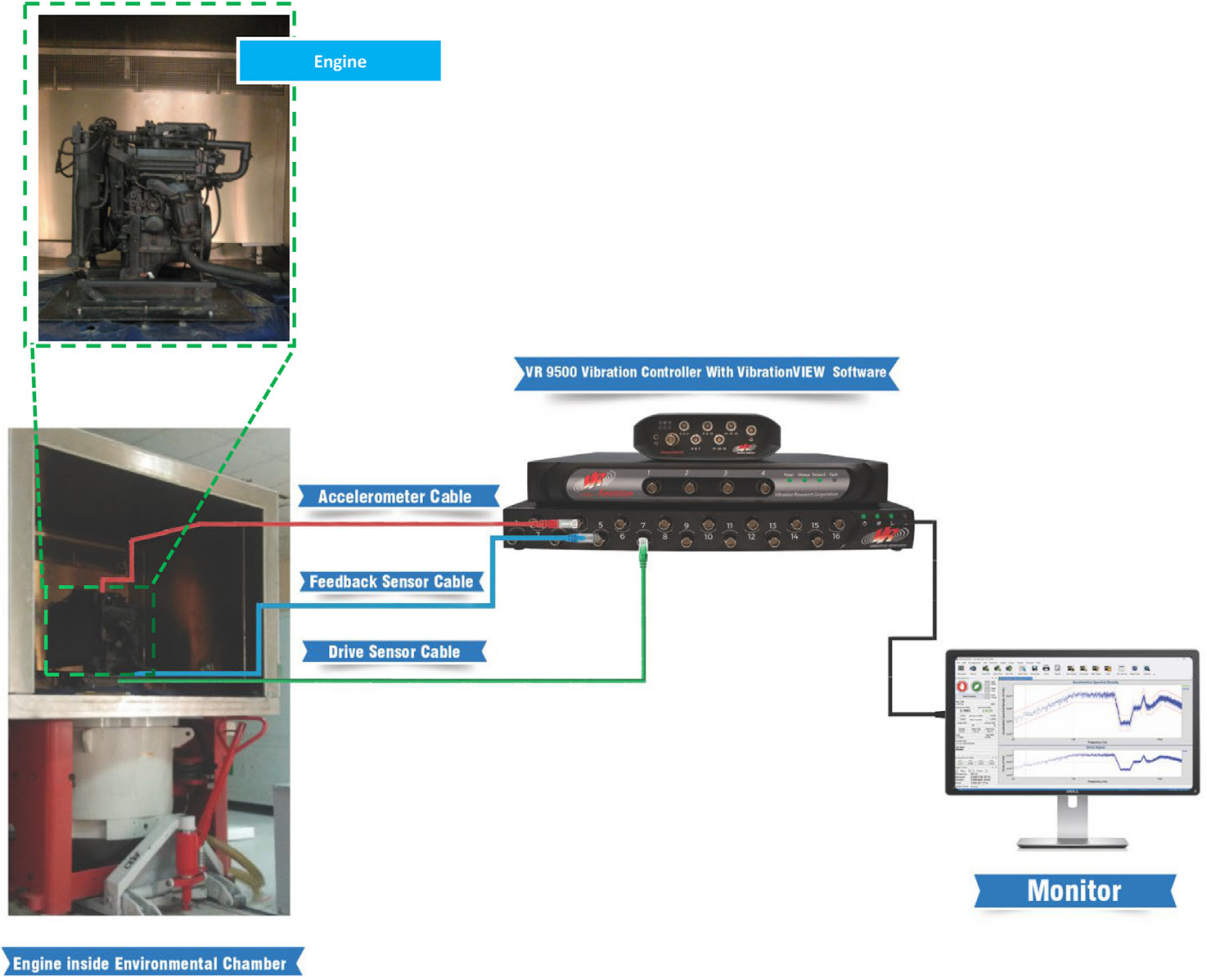
The experimental structure.

**Experimental Duration and Condition Simulation**: The engine was subjected to randomly assigned climatic and operating conditions based on the Full-Factorial design method, resulting in 45 data recordings. The engine ran continuously for approximately eight hours to simulate real-world conditions, with each climatic condition tested at different speeds for about two minutes. The controller generated a signal that was amplified and sent to the vibrator, with control channels monitoring and providing feedback on the signals. The experimental setup is illustrated in Fig. 15.

### 4.6 Calibration and standards

**Calibration Process**: All measurement equipment—tri-axial accelerometer, tachometer, humidity sensors, and thermocouples—was calibrated before each set of experiments. Calibration was done according to the manufacturer's instructions to ensure precision. The calibration ensured that all data collected during the experiment accurately reflected the actual operating conditions.

**Standard Followed**: The experiments were conducted in accordance with MIL-STD-810G guidelines, which provide standardized procedures for testing equipment under different environmental conditions. MIL-STD-810G is a military standard established by the U.S. Department of Defense (DoD) to assess the durability and environmental ruggedness of devices under test (DUT) when subjected to a wide range of operational and environmental conditions [17]. It is widely adopted across industries such as aerospace, electronics, transportation, and consumer products to certify the durability and reliability of products in harsh environments [17]. The purpose of this standard is to ensure that equipment—whether military or commercial—can withstand physical and environmental stresses likely to be encountered during its life-cycle, including extreme temperatures, vibrations, shocks, and humidity.

When combined, these stressors can lead to equipment failures. The objective of this standard is to provide guidance on the application of environmental tailoring throughout the material acquisition cycle. For this study, we selected MIL-STD-810G Method 520.3, titled 'Temperature, Humidity, Vibration, and Altitude,' which evaluates the combined effects of temperature, humidity, altitude, and vibration on airborne electronics and electro-mechanical equipment, focusing on integrity, safety, and performance during operation. Although this method was designed for airborne equipment, it is equally applicable to ground platforms, with the exception of altitude considerations for ground vehicles. In this test, the combined effects of temperature, humidity, and vibration were studied, as these are the conditions most likely to be encountered by commercial vehicles. After selecting the method and relevant procedure, the environmental parameters—such as humidity, temperature, and vibration—under which the DUT (vehicle engine) would be deployed or operated were determined. This step is part of a test cycle in which the DUT will undergo various performance profiles under different climatic conditions. MIL-STD-810G allows for tailoring environmental conditions to closely match the real-world situations the DUT is likely to face.”

This ensured that the data was collected under consistent and repeatable conditions, enhancing the reliability of the dataset. The **MIL-STD-810G** standard was specifically chosen to simulate the harsh environmental conditions that bearings might face during their operational life, making the dataset highly valuable for predictive maintenance and reliability analysis.

### 4.7 Testing procedure

**Fig. 16:**
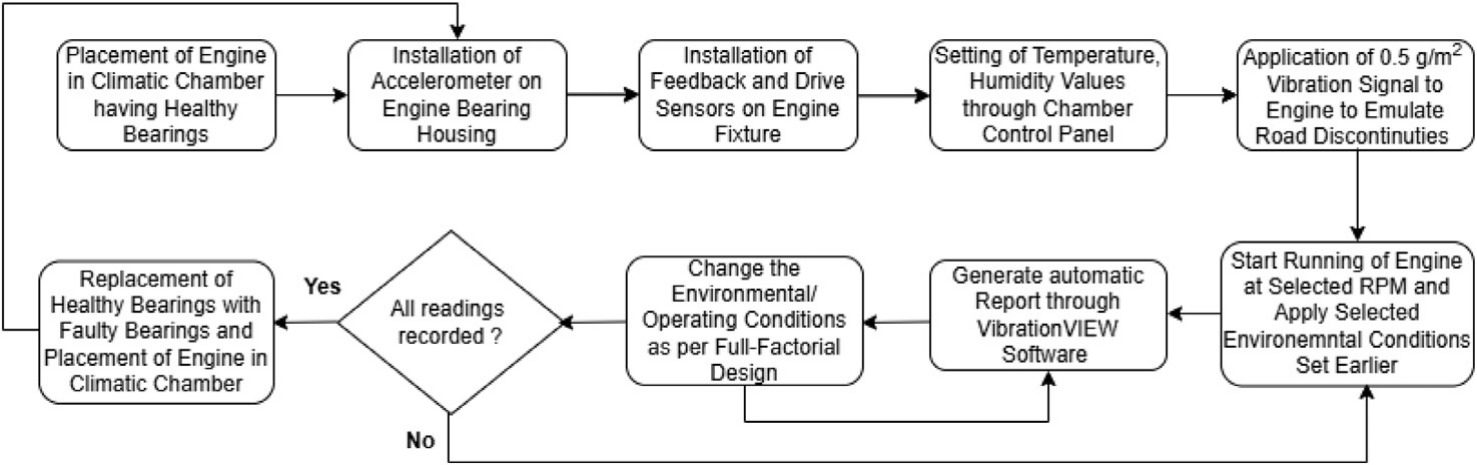
Procedure for Data Collection.

The application of statistical methods and mathematical analysis significantly enhances experimental efficiency and facilitates the extraction of meaningful insights from the collected data [18]. For successful experimentation, it is crucial for researchers to identify the factors influencing output or response parameters. Understanding these critical variables is essential for ensuring accurate results. The Design of Experiments (DOE) is valuable for identifying key input variables that affect process variability [18]. Many processes involve complex interactions, and overlooking these can impede a thorough understanding [18]. DOE, in addition, enables the exploration of interactions between different parameters. The testing procedure for data collection is shown in Fig. 16.”

For data acquisition, we selected five temperature levels (−10 °C, 0 °C, 15 °C, 30 °C, and 45 °C), three humidity levels (0%, 50%, and 100%), and three engine rotational speeds (1000 RPM, 1500 RPM, and 2000 RPM) to replicate the real-world environmental and operating conditions for the vehicle engine. The Root Mean Square (RMS) value of the acquired signal was used as the dependent variable. The rationale behind focusing on low-engine, low-speed vehicles, and the climatic and traffic conditions in regions like Far-East Asia, South-Asia, South-East Asia, Latin America, and Africa is elaborated in Table 4:

**Table 4:** Input Parameters for the Experiments.

Input Parameters	Values/ Range of Parameters	Rationale for Selecting Parameters
Temperature	−10, 0, 15, 30, 45(°C)	These temperatures simulate real-world operating environments, from freezing to hot climates, prevalent in regions such as Far-East Asia, South Asia, and Latin America. In addition, the range aligns with MIL-STD-810G standards to represent the harsh conditions typical of these regions.
Humidity	0, 50, 100 (%)	Selected to cover a wide range of moisture levels from dry to extremely humid, representing the climates found in South-East Asia, Africa, and Latin America.
Engine RPM	1000, 1500, 2000 (RPM)	The RPM values have been chosen based on the operation of smaller engines used in urban environments with stop-and-go traffic regions such as South-Asia, Far-east Asia, and Africa where traffic congestion and low-speed driving are common. Lower RPM engines are more fuel-efficient, a key concern in these regions.
Vibration Signal	0.5 g m/s^2^	A continuous vibration signal of 0.5 g m/s^2^, which is half the value of allowable vibration for military vehicles as recommended by the MIL-STD-810G standard, has been applied to device under test (DUT)/ vehicle engine to simulate the surface and road discontinuities. while moving on the road.
Bearing Condition	Healthy, Faulty	Testing both healthy and faulty journal bearings allows for robust fault detection analysis under real-world conditions.

As the real world vibrations are often unpredictable/ random, a continuous random vibration signal of 0.5 g m/s², half the permissible level for military vehicles as per MIL-STD-810G, was applied to the DUT to simulate surface and road discontinuities [17]. This signal mimics the road conditions experienced by a vehicle during operation. Although MIL-STD-810G is designed for Department of Defense(DoD) applications, it is adaptable for commercial use as well [17]. The standard establishes laboratory test procedures to replicate environmental effects on materials, focusing on customizing design and test limits to the conditions a material will encounter throughout its service life.

Following the selection of the method and procedure, the specific levels of environmental parameters—such as humidity, temperature, and vibration—are defined for the DUT (vehicle engine). This step is integral to the test cycle, during which the DUT is subjected to various profiles under different climatic conditions. In addition, MIL-STD-810G allows for the adaptation of environmental conditions to reflect the real-world scenarios the DUT will encounter, while also accounting for the constraints of the testing chamber.

**Fig. 17:**
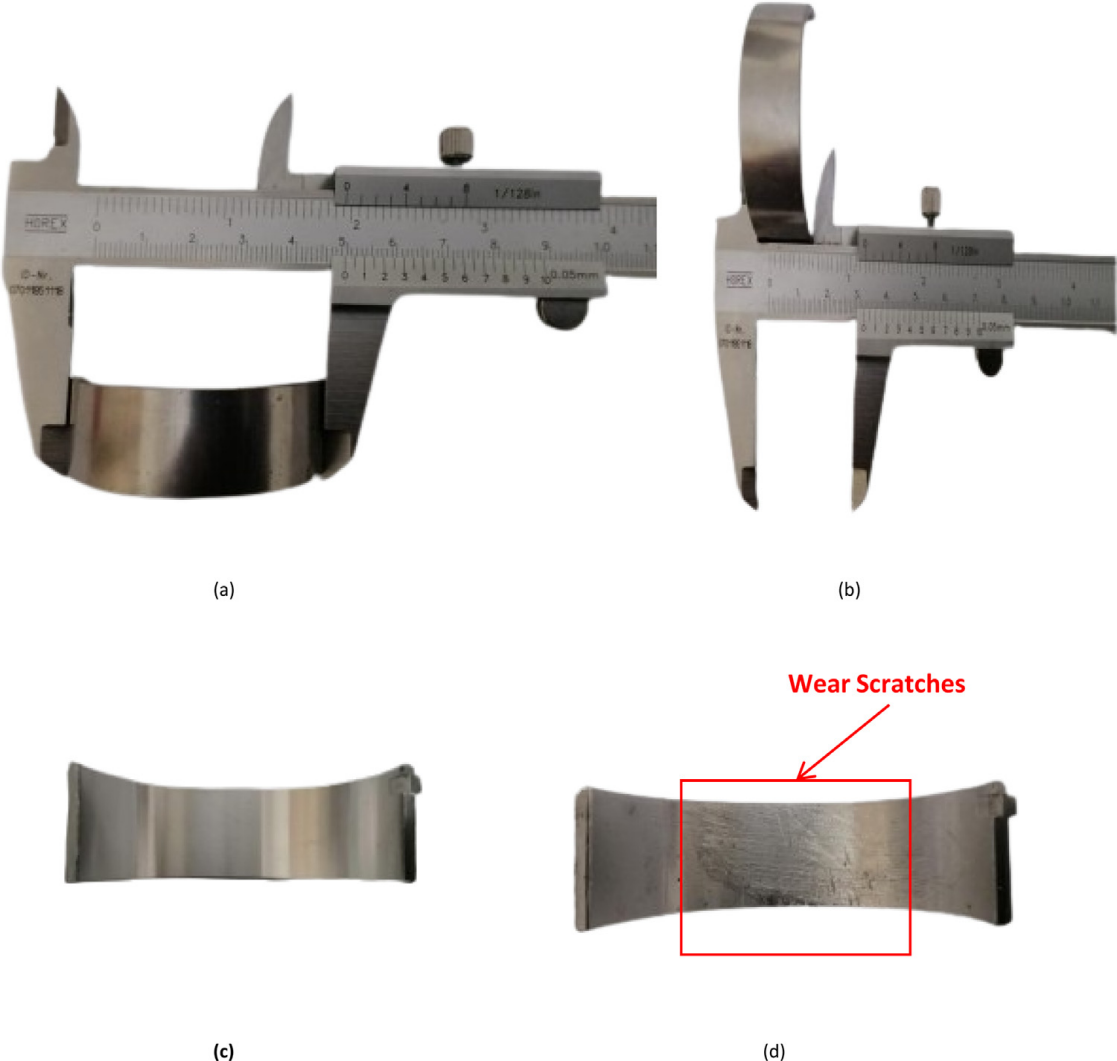
(a) Bearing Outer Diameter (b) Bearing Width. (c) Healthy Bearing (d) Faulty Bearing.

Minitab® v18 statistical software was employed to determine the optimal number of experiments [19]. Forty-five experiments for both healthy and faulty bearings shown in Fig. 17 were conducted using Full-Factorial design, as detailed in Tables 5 and 6. These experiments were repeated four times in the same order to cater fore any incorrect recording of data/ RMS value.

**Table 5:** Experimental design matrix (healthy bearings).

Standard Order	Run Order	Input Parameters		
		Temperature (°C)	Humidity (%)	Engine Rotation Speed (RPM)
1	38	45	0	1500
2	19	15	0	1000
3	42	45	50	2000
4	43	45	100	1000
5	14	0	50	1500
6	9	−10	100	2000
7	13	0	50	1000
8	40	45	50	1000
9	45	45	100	2000
10	4	−10	50	1000
11	23	15	50	1500
12	6	−10	50	2000
13	17	0	100	1500
14	25	15	100	1000
15	44	45	100	1500
16	41	45	50	1500
17	18	0	100	2000
18	29	30	0	1500
19	30	30	0	2000
20	24	15	50	2000
21	20	15	0	1500
22	35	30	100	1500
23	33	30	50	2000
24	7	−10	100	1000
25	11	0	0	1500
26	28	30	0	1000
27	15	0	50	2000
28	1	−10	0	1000
29	37	45	0	1000
30	27	15	100	2000
31	31	30	50	1000
32	16	0	100	1000
33	8	−10	100	1500
34	22	15	50	1000
35	5	−10	50	1500
36	3	−10	0	2000
37	39	45	0	2000
38	2	−10	0	1500
39	21	15	10	2000
40	10	0	0	1000
41	32	30	50	1500
42	26	15	0	1500
43	36	30	0	2000
44	12	0	0	2000
45	34	30	100	1000

**Table 6:** Experimental design matrix (faulty bearings).

Standard Order	Run Order	Input Parameters		
		Temperature (°C)	Humidity (%)	Engine Rotation Speed (RPM)
1	10	45	0	1000
2	1	15	0	1500
3	45	45	50	2000
4	40	45	100	1000
5	35	0	50	1500
6	9	−10	100	2000
7	2	0	50	1000
8	44	45	50	1000
9	39	45	100	2000
10	8	−10	50	1000
11	3	15	50	1500
12	43	−10	50	2000
13	38	0	100	1000
14	34	15	100	1500
15	7	45	100	1500
16	4	45	50	1500
17	42	0	100	2000
18	37	30	0	2000
19	33	30	0	1500
20	30	15	50	2000
21	6	15	0	1500
22	5	30	100	1500
23	41	30	50	2000
24	36	−10	100	1000
25	32	0	0	1000
26	29	30	0	1500
27	26	0	50	2000
28	21	−10	0	1000
29	24	45	0	1000
30	19	15	100	2000
31	31	30	50	1000
32	17	0	100	1500
33	13	−10	100	1000
34	15	15	50	1000
35	12	−10	50	1500
36	28	−10	0	2000
37	23	45	0	2000
38	18	−10	10	1500
39	11	15	0	2000
40	22	0	0	1000
41	27	30	50	1500
42	16	15	100	2000
43	14	30	100	1500
44	20	0	0	2000
45	25	30	100	1000

**Fig. 18:**
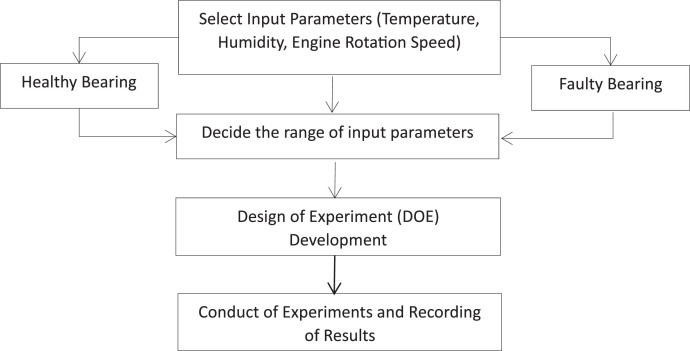
Flowchart representing full-factorial design implementation.

Despite its higher cost and complexity, Full-Factorial Design provided comprehensive insights into engine behavior under varying conditions, justifying its use over Fractional Factorial Design [20]. The process of implementing Full-Factorial Design is illustrated in Fig. 18.

## Limitations

The current dataset is focused on smaller engines typically used in urban environments, where low-speed driving and low-RPM operation are common. While this dataset includes data for healthy bearings and inner ring faults, collecting data for other fault types, such as outer ring faults and combined faults, remains a goal for future research. Additionally, gathering high-RPM data for larger engines is planned to provide a broader understanding of bearing performance. However, the resource-intensive nature of operating climatic chambers and engine setups limits the scope of data collection in this phase.

## Ethics Statement

The authors have read and follow the ethical requirements for publication in Data in Brief and confirming that the current work does not involve human subjects, animal experiments, or any data collected from social media platforms.

## CRediT Author Statement

**Muhammad Noman Riaz.:** Conceptualization, Methodology, Data Acquisition, Formal Analysis**,** Software, Resources, Writing-Original Draft; **Amir Hamza:** Project administration, Funding Acquisition, Supervision and Validation; **Hamid Jabbar:** Supervision, Reviewing and Editing; **Manzar Abbas:** Methodology, Supervision, Reviewing and Editing; **Mohsin Islam Tiwana:** Supervision, Reviewing and Editing.

## Data Availability

Mendeley DataVibration dataset of main journal bearings in internal combustion engines under diverse climatic and varying operating conditions (Original data).

## References

[1] Nithin S.K., Hemanth K., Shamanth V. (2021). A review on combustion and vibration condition monitoring of IC engine. Mater. Today Proc..

[2] Mohamed E.S. (2018). Performance analysis and condition monitoring of ICE piston-ring based on combustion and thermal characteristics. Appl. Therm. Eng..

[3] Laubichler C., Kiesling C., Marques da Silva M., Wimmer A., Hager G. (2022). Data-Driven sliding bearing temperature model for condition monitoring in internal combustion engines. Lubricants.

[4] Heywood J. (2018).

[5] Kalghatgi G., Levinsky H., Colket M. (2018). Future transportation fuels. Prog. Energy Combust. Sci..

[6] Antoni J., Daniere J, Guillet F (2002). Effective vibration analysis of IC engines using cyclostationarity. Part I-A methodology for condition monitoring. J. Sound Vibr..

[7] Lee S., Kim T., Kim T. (2024). Multi-domain vibration dataset with various bearing types under compound machine fault scenarios. Data Brief.

[8] Khalifa R.M., Yacout S., Bassetto S., Shaban Y. (2023). Experimental vibration data collected for a belt drive system under different operating conditions. Data Brief.

[9] Jung W., Yun S.-H., Park Y.-H. (2024). Vibration, and temperature run-to-failure dataset of ball bearing for prognostics. Data Brief.

[10] Sehri M., Dumond P. (2024). University of Ottawa constant and variable speed electric motor vibration and acoustic fault signature dataset. Data Brief.

[11] Kumar D., Mehran S., Shaikh M.Z., Hussain M., Chowdhry B.S., Hussain T. (2022). Triaxial bearing vibration dataset of induction motor under varying load conditions. Data Brief.

[12] Sehri M., Dumond P., Bouchard M. (2023). University of Ottawa constant load and speed rolling-element bearing vibration and acoustic fault signature datasets. Data Brief.

[13] Bruinsma S., Geertsma R.D., Loendersloot R., Tinga T. (2024). Motor current and vibration monitoring dataset for various faults in an E-motor-driven centrifugal pump. Data Brief.

[14] Marshall L., Jensen D. (2023). Dataset of single and double faults scenarios using vibration signals from a rotary machine. Data Brief.

[15] Ismail M.A.A., Windelberg J., Bierig A., Bravo I., Arnaiz A. (2023). Ball bearing vibration data for detecting and quantifying spall faults. Data Brief.

[16] Arpa L., Gabrielli A., Battarra M., Mucchi E. (2024). University of Ferrara run-to-failure vibration dataset of self-aligning double-row ball bearings. Data Brief.

[17] MIL-STD-810G Working Group, ed. (2008), *MIL-STD-810G, Department of Defense Test Method Standard - Environmental Engineering Considerations and Laboratory Tests.*

[18] Myers RH, Montgomery DC (2002).

[19] Patil M.S., Mathew J., Rajendrakumar P.K., Karade S. (2010). Experimental studies using response surface methodology for condition monitoring of ball bearings. J. Tribol..

[20] Nithin S.K., Hemanth K., Shamanth V. (2021). A review on combustion and vibration condition monitoring of IC engine. Mater. Today Proc..

